# Impact of financial incentives on viral suppression among adults initiating HIV treatment in Tanzania: a hybrid effectiveness–implementation trial

**DOI:** 10.1016/S2352-3018(24)00149-8

**Published:** 2024-08-01

**Authors:** Prosper F Njau, Emmanuel Katabaro, Solis Winters, Amon Sabasaba, Kassim Hassan, Babuu Joseph, Hamza Maila, Janeth Msasa, Carolyn A Fahey, Laura Packel, William H Dow, Nicholas P Jewell, Nzovu Ulenga, Natalino Mwenda, Sandra I McCoy

**Affiliations:** National AIDS, STIs and Hepatitis Control Programme, Ministry of Health, Dodoma, Tanzania; Health for a Prosperous Nation, Dar es Salaam, Tanzania; School of Public Health, University of California, Berkeley, CA, USA; Health for a Prosperous Nation, Dar es Salaam, Tanzania; Health for a Prosperous Nation, Dar es Salaam, Tanzania; Health for a Prosperous Nation, Dar es Salaam, Tanzania; Health for a Prosperous Nation, Dar es Salaam, Tanzania; Health for a Prosperous Nation, Dar es Salaam, Tanzania; School of Public Health, University of California, Berkeley, CA, USA; School of Public Health, University of Washington, Seattle, WA, USA; School of Public Health, University of California, Berkeley, CA, USA; School of Public Health, University of California, Berkeley, CA, USA; School of Public Health, University of California, Berkeley, CA, USA; London School of Hygiene & Tropical Medicine, London, UK; Management and Development for Health, Dar es Salaam, Tanzania; Rasello, Dar es Salaam, Tanzania; School of Public Health, University of California, Berkeley, CA, USA

## Abstract

**Background:**

Small incentives could improve engagement in HIV care. We evaluated the short-term and longer-term effects of financial incentives for visit attendance on viral suppression among adults initiating antiretroviral therapy (ART) in Tanzania.

**Methods:**

In a type 1 hybrid effectiveness–implementation study, we randomised (1:1) 32 primary care HIV clinics in four Tanzanian regions to usual care (control group) or the intervention (usual care plus ≤6 monthly incentives ART (<30 days) who owned a mobile phone and had no plans to transfer to another facility were eligible. The primary outcome was retention on ART with viral suppression (<1000 copies per mL) at 12 months. Secondary outcomes included retention on ART with viral suppression at 6 months and viral suppression at 6 months and 12 months using a lower threshold (<50 copies per mL). Intent-to-treat analysis and a cluster-based permutation test were used to evaluate the effect of financial incentives on outcomes. This trial is registered with ClinicalTrials.gov, NCT04201353, and is completed.

**Findings:**

Between May 28, 2021, and March 8, 2022, 1990 participants (805 male and 1185 female) were enrolled in the study. 1059 participants were assigned to the intervention group and 931 participants were assigned to the control group. Overall, 1536 (88%) participants at 6 months and 1575 (83%) at 12 months were on ART with viral suppression. At 12 months, 6 months after the intervention ended, 866 (85%) participants in the intervention group compared with 709 (81%) in the control group had viral loads less than 1000 copies per mL (adjusted risk difference [aRD] 4.4 percentage points, 95% CI −1.4 to 10.1, permutation test p=0.35). At 6 months, 858 participants (90%) in the intervention group were on ART with viral loads less than 1000 copies per mL compared with 678 (86%) in the control group (aRD 5.1 percentage points, 95% CI 1.1 to 9.1, permutation test p=0.06). Effects were larger at 6 months and 12 months with the lower threshold for viral suppression, and there was significant effect heterogeneity by region. Adverse events included 106 deaths (56 in the control group and 50 in the intervention group), none related to study participation.

**Interpretation:**

Short-term incentives for visit attendance had modest, short term benefits on viral suppression and did not harm retention or viral suppression after discontinuation. These findings suggest the need to understand subgroups who would most benefit from incentives to support HIV care.

**Funding:**

National Institute of Mental Health.

## Introduction

The biomedical advances in HIV care over the last decade have coincided with increasing recognition of the importance of human behaviour to realise their potential. For example, highly effective tools such as pre-exposure prophylaxis (PrEP) for HIV prevention, or antiretroviral therapy (ART) for HIV treatment, rely on behaviours like daily adherence that can determine whether efficacy observed in trials translates to infections averted and reductions in population-level morbidity and mortality.^1^ Decades of research have revealed that HIV care-seeking behaviour is influenced not only by factors at the level of individual, household, and community, but also unconscious factors that together can erode connections to HIV prevention and care, undermine adherence, and ultimately lead to disengagement.^2^ Consequently, despite enormous progress, approximately one in four people living with HIV in eastern and southern Africa in 2021 was not virally suppressed,^3^ heightening their risk of morbidity and mortality and onward transmission, and underscoring the need for continued innovation.

Recognising the importance of external influences on HIV care engagement, including structural factors like poverty and food insecurity,^4^ as well as unconscious determinants like motivation, we embarked on a multiphase process to design and evaluate a behavioural intervention to support people initiating ART in Tanzania to achieve and maintain viral suppression. Informed by the Multiphase Optimization Strategy framework^5^ that was designed to accelerate the development and roll-out of effective, optimised interventions, we did a series of experiments to iteratively prepare a financial incentive intervention and implementation strategy for large-scale evaluation and future scaling up (if effective).^6^ A proliferation of studies has revealed that when tailored to the desired behaviour and context, financial and non-financial incentives can increase HIV testing, retention in care, and adherence to ART.^7–10^ The growing evidence base for these approaches has increased calls to bring effective approaches to scale.^11,12^

Our first study (preparation phase) was a three-arm, randomised, non-inferiority trial comparing different intervention approaches to support people living with HIV starting ART, finding that small, monthly financial incentives for visit attendance bolstered ART possession to levels similar to food support, with lower costs and a higher preference for cash (versus food) among beneficiaries.^13^ We did a series of qualitative studies to understand the pathways of effect and safety;^14,15^ as well as a mixed methods implementation study to develop and refine a scalable mHealth system for clinic-based implementation.^16^ We then evaluated two versions of the intervention approach that varied the size of the financial incentive in a separate three-arm trial at four clinics, using viral suppression as the outcome (optimisation phase).^17^ The final version of the financial incentive intervention (22 500 Tanzanian Shillings [TZS], about $10 USD, per month for up to 6 months, conditional on visit attendance) achieved a significant 13.0 percentage point improvement in viral suppression over usual care (control group) at 6 months.

Now we report on the intervention’s effectiveness on retention on ART with viral suppression in four regions (evaluation phase).^18^ This trial was designed to address outstanding gaps in the HIV literature, such as whether the pilot study results are replicated at a larger scale, and whether incentive interventions have a beneficial or harmful effect on viral suppression after their removal.

## Methods

### Study design and participants

We did a type 1 hybrid effectiveness–implementation cluster-randomised trial to evaluate the effectiveness of short-term financial incentives on viral suppression and retention in care among adults initiating ART at 32 HIV primary care clinics across four regions of Tanzania.^18^ Ethical approval was obtained from the Institutional Review Board at the University of California, Berkeley, CA, USA and the National Institute for Medical Research in Dar es Salaam, Tanzania. The study was overseen by a data safety and monitoring board, and registered with ClinicalTrials.gov, NCT04201353.

Government health facilities providing HIV primary care in Geita, Kagera, Mwanza, or Shinyanga regions were eligible if they used an electronic medical record database, were within 100 km of a city centre, were at least 15 km from another study clinic, and had an average of 65 or more new ART initiates per quarter and no fewer than 35 in any single quarter in 2019, when the study was initiated, to ensure there would be an adequate number of potential study participants. Eligible study participants were adults (aged ≥18 years) initiating ART (<30 days on ART at enrolment) who owned a mobile phone and were not planning to transfer to another facility within 12 months. Written informed consent was obtained from all participants.

### Randomisation and masking

Overall, 32 eligible health facilities (eight per region) were randomly selected and randomised 1:1 to usual care or to the intervention using covariate constrained randomisation with clinic-level characteristics and stratification by region. Randomisation was done via a random sequence generator in R. Given the nature of the intervention, clinic staff, participants, and analysts were not masked.

After study launch, two control facilities met the a priori threshold in the protocol for replacement due to slow enrolment and were replaced with eligible, randomly selected clinics in the same regions. Data collected for participants at the two replaced facilities were not included in the analysis.

### Procedures

All participants in the study, regardless of study group, received the standard of care as provided by HIV primary care clinics in Tanzania. In addition, all clinics in the study enrolled and registered patients using a custom mHealth system (implementation model) with biometric identification verification, implemented by clinical staff.^16^ Study participants enrolled in the mHealth system and subsequently checked-in for each visit first at the registration desk with the fingerprint scanner and again in the pharmacy for ART dispensing. The system tracked patient visits, upcoming appointments, ART dispensing, cash disbursement (intervention clinics) and HIV viral load monitoring, with pop-up reminders for clinic staff. The mHealth system sent all participants (intervention and control) SMS visit reminders.

Participants enrolled in intervention sites additionally received the opportunity to receive up to 6 consecutive monthly (≥25 days apart) financial incentives of 22 500 TZS each (about US$10). Incentives were conditional on visit attendance during the first 6 months of the study period, which intentionally aligned with Tanzania’s monthly visit schedule in the first 6 months of ART. Cash was automatically disbursed to participants’ mobile banking accounts when they checked into the mHealth system for an eligible visit (ie, intervention participant, ≥25 days since their last incentive, ≤6 total incentives, and ≤183 days since enrolment). The incentive amount of 22 500 TZS was based on the results of the optimisation phase of the study.^17^ In addition, we consulted extensively with our Ministry of Health and clinical collaborators to ensure that the amount was potentially scalable should the intervention be effective.^18^ The decision to incentivise visit attendance was made after consultation with HIV caregivers and Ministry of Health stakeholders, who wanted to motivate engagement in the spectrum of clinical activities crucial for care management, such as clinical evaluation, adherence counselling, and immunological and virological monitoring (when applicable). The use of incentives to motivate behaviour change is supported by three theoretical paradigms: self-determination theory, which focuses on engaging in a behaviour because of an anticipated reward;^19^ behavioural economics, which leverages biases in human decision making to motivate engagement in particular behaviours;^20^ and microeconomic theory, which states that people will obtain more of a lower priced good or service than a higher priced one.^21^ The use of incentives in this setting leverages these theories through motivating continuation of care through a promised reward to offset present-biased preferences,^7^ leveraging the behavioural economics concept of salience by tying the incentive directly to ART pick-up, and the idea that the total cost of a clinical visit is reduced or partly offset by the incentive.

At enrolment, 6 months, and 12 months, participants completed a survey implemented via Qualtrics software including demographic and health information, experiences in HIV care, and perceptions of the intervention (if intervention group). Visits dates, pharmacy records, and incentive disbursement data were recorded directly by clinic staff in the mHealth system. Virological monitoring was done 6 months and 12 months after initiation of ART, as per national guidelines, and was quantified with the Abbott RealTime HIV-1 viral load assay (HIV-1; Abbott Molecular, Des Plaines, IL, USA, Cobas HIV-1 Quantitative nucleic acid test kit for C4800 systems (Roche, Branchburg, NJ, USA), and the Xpert HIV-1 viral load test (Cepheid, Sunnyvale, CA, USA). Viral load results were automatically imported into the mHealth system via an Application Programming Interface. All mHealth data were periodically triangulated with the national HIV care database, the laboratory database and paper medical records to ensure completion.

Given that silent transfers (patient transfers to other clinics that are unknown to the sending facility or research team) have the potential to introduce bias, we used gold-standard procedures for extensive tracing of all participants missing 12-month viral load results. In brief, this included: three phone calls per day for 3 consecutive days using all available phone numbers; SMS messages; and, for those not contacted after nine total calls, linkage to home-based care for three in-person contact attempts. Tracing was considered complete if a participant was reached or if all nine phone and three in-person attempts had been completed.

To understand the degree to which the intervention was implemented as intended, we defined intervention fidelity as the proportion of eligible visits at each clinic for which the incentive was successfully sent to the participant within 48 h. Intervention fidelity was set to 1 for all control clinics, since no incentives were sent to these participants, as intended based on the implementation design.

### Outcomes

The primary outcome was a binary variable of retention on ART with HIV viral suppression (<1000 copies per mL) at 12 months. In addition to participants on ART with viral suppression, the denominator included the since the last missed visit; and those who have died. Participants retained on ART with an unmeasured viral load at 6 months or 12 months were considered missing and not included in the analyses. At the suggestion of collaborators at the Ministry of Health, we additionally evaluated the primary outcome with viral suppression defined as less than 50 copies per mL; this analysis was not prespecified.

Prespecified secondary outcomes were retention on ART with viral suppression (<1000 copies per mL) at 6 months, retention on ART at 6 and 12 months, viral suppression (<1000 copies per mL) among those retained on ART at 6 months and 12 months, and proportion of scheduled visits attended within 4 days (the incentivised behaviour) at 6 months and 12 months.

Viral loads specimens taken at 5–8 months (median 183 days) and 8–16 months (median 374 days) after ART initiation were considered valid 6 month and 12 month viral loads, respectively. If more than one viral load result was recorded within the window, the specimen collected closest to the 6-month or 12-month mark was used.

### Statistical analysis

To detect a minimum effect of 11 percentage points on our primary outcome with 80% power, estimated ICC of 0.05, and 10% inflation to account for any issues during implementation, we set a target sample size of 1984 participants (62 participants × 32 clinics).^18^

Per our pre-specified analysis plan (osf.io/ces65), we did an intent-to-treat (ITT) analysis and a cluster-based permutation test to evaluate the effect of financial incentives on primary and secondary outcomes. Linear probability models were used to estimate ITT risk differences. Our primary model included region fixed effects (model 1) to account for the stratified study design. We also reported estimates adjusting for additional covariates. Model 2 included clinic-level characteristics used in constrained randomisation (facility type, log average ART initiates per quarter in 2019, distance to a major city, and proximity [<5 km] to a major road), and model 3 additionally adjusted for participant age, gender, and WHO clinical stage at baseline (with categories for stages 1–4 or missing). Clustered sandwich estimators were used to adjust SEs for clustering at the clinic level.

We also did additional analyses, estimated using the model 1 specification. To assess differences in the short-term (6-month) and longer-term (12-month) effects of the intervention on the primary outcome, we constructed a repeated measures linear probability model with an interaction between study group and time. We evaluated the effect of clinic-level intervention fidelity on the primary outcomes at 6 months and 12 months using a two-stage least-squares instrumental variable model with random assignment to intervention group as the instrument and intervention fidelity as the treatment variable. Subgroup analyses were done by region, facility type, gender, age, and household wealth.

In addition, we did exploratory analyses to elucidate some of the study findings. We assessed intervention effects on ART dispensing and visit attendance among those retained on ART at 6 months and 12 months to uncover any potential differences in adherence among those in care during the study period. Additionally, to statistically assess the observed difference in intervention effect in Kagera region relative to the other regions, we constructed a model with an interaction term between study group and an indicator for Kagera facilities. We then evaluated the effect of the intervention on subsamples of data excluding all Kagera facilities (appendix 2 p 1), and excluding only two specific Kagera facilities that had a higher proportion of migrant workers (appendix 2 p 2).

As a sensitivity analysis, we reran the primary outcome models using multiple imputation to impute viral suppression status for the subset of patients who were retained on ART but were missing viral load data at 6 months or 12 months, and thus excluded from the main results. Multiple imputation by chained equations was conducted using the mice package in R. Missing outcome data were imputed 20 times using a logistic regression model with the same set of covariates included in model 3. Pooled estimates are presented in appendix 2 (p 3).

All analyses were done in R Studio. For interpretability, risk differences are scaled by 100 and expressed as percentage-point differences in text and associated figures.

### Role of the funding source

The funder of the study had no role in study design, data collection, data analysis, data interpretation, or writing of the report.

## Results

**Figure 1:**
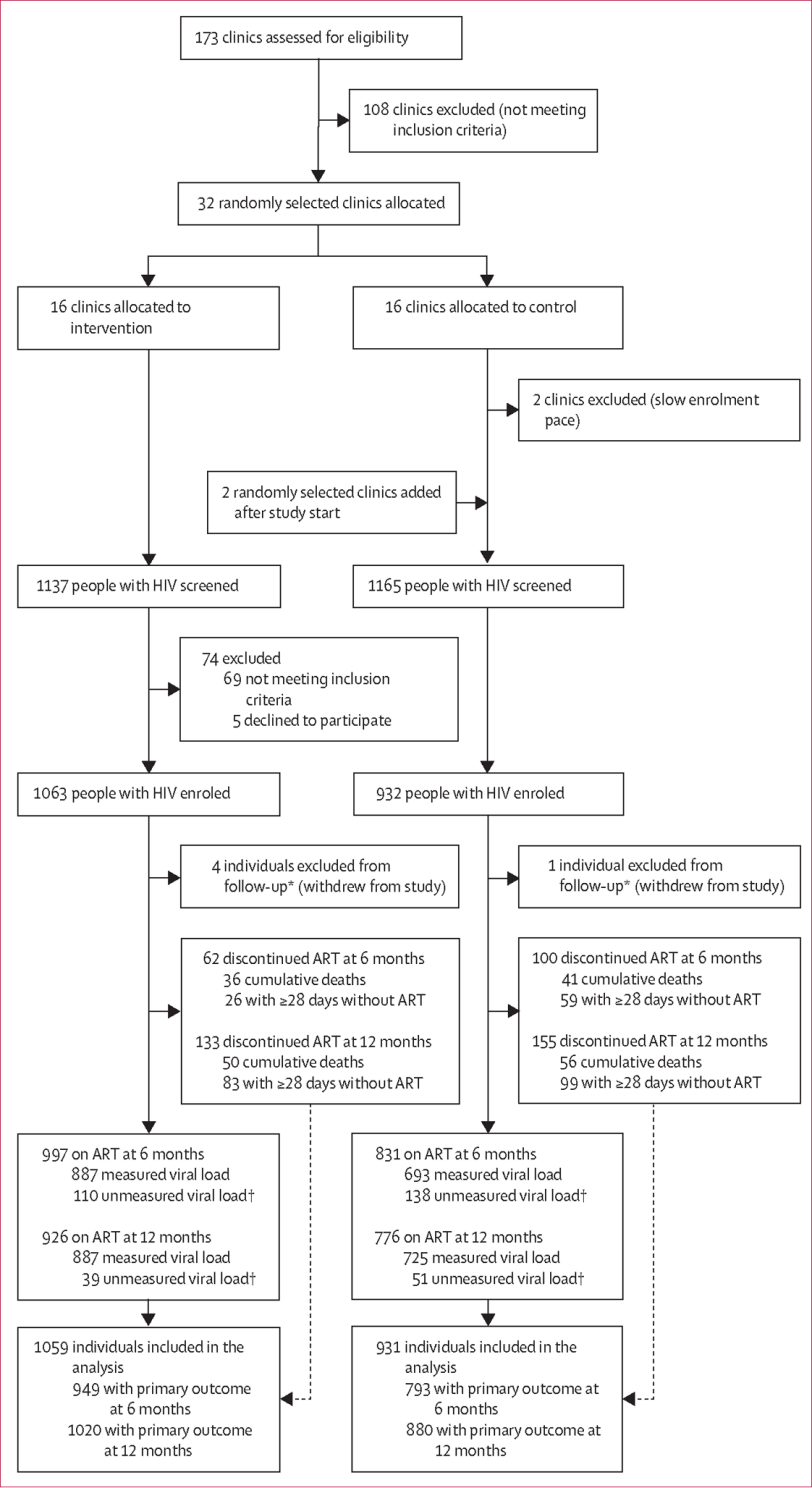
Trial profile. ART=antiretroviral therapy. *Loss to follow-up is not applicable in our study because traditional indicators of follow-up such as death or discontinued treatment are part of the primary outcome definition; instead, we provide details on trial status at 6 months and 12 months. †The primary outcome includes all participants retained on ART with a measured viral load or who discontinued ART; participants on ART with an unmeasured viral load were considered missing from the primary outcome and not included in associated analyses.

Between May 28, 2021, and March 7, 2022, a total of 32 randomly selected HIV primary care clinics (nine dispensaries, 13 health centres, and ten hospitals) were randomised (1:1) to the intervention or usual care, from which 1995 participants were enrolled into the study; follow-up continued until July 14, 2023 (figure 1). Five participants withdrew after enrolment, leaving 1990 total participants in the analytic sample (mean 62 per site; range 36–77 per site; 1059 in the intervention group and 931 in the control group). At 12 months, 1702 (86%) participants were retained on ART, of which 1612 (95%) had HIV viral load results; 182 (9%) discontinued ART and 106 (5%) had died. 90 participants retained on ART but with no viral load measure were considered missing from the primary outcome; 248 were considered missing from the outcome at 6 months.

Participants were, on average, 37 years old and on ART for 3.5 days (99.8% on dolutegravir-based regimens) at baseline. 1185 (60%) identified as female, 1193 (61%) were married or partnered, 1133 (57%) had completed primary education, and 1231 (63%) were employed. 1317 participants (67%) screened positive for symptoms of depression and 1201 (61%) for symptoms of anxiety. Baseline characteristics were mostly balanced between the intervention and control group (table 1).

**Figure 2:**
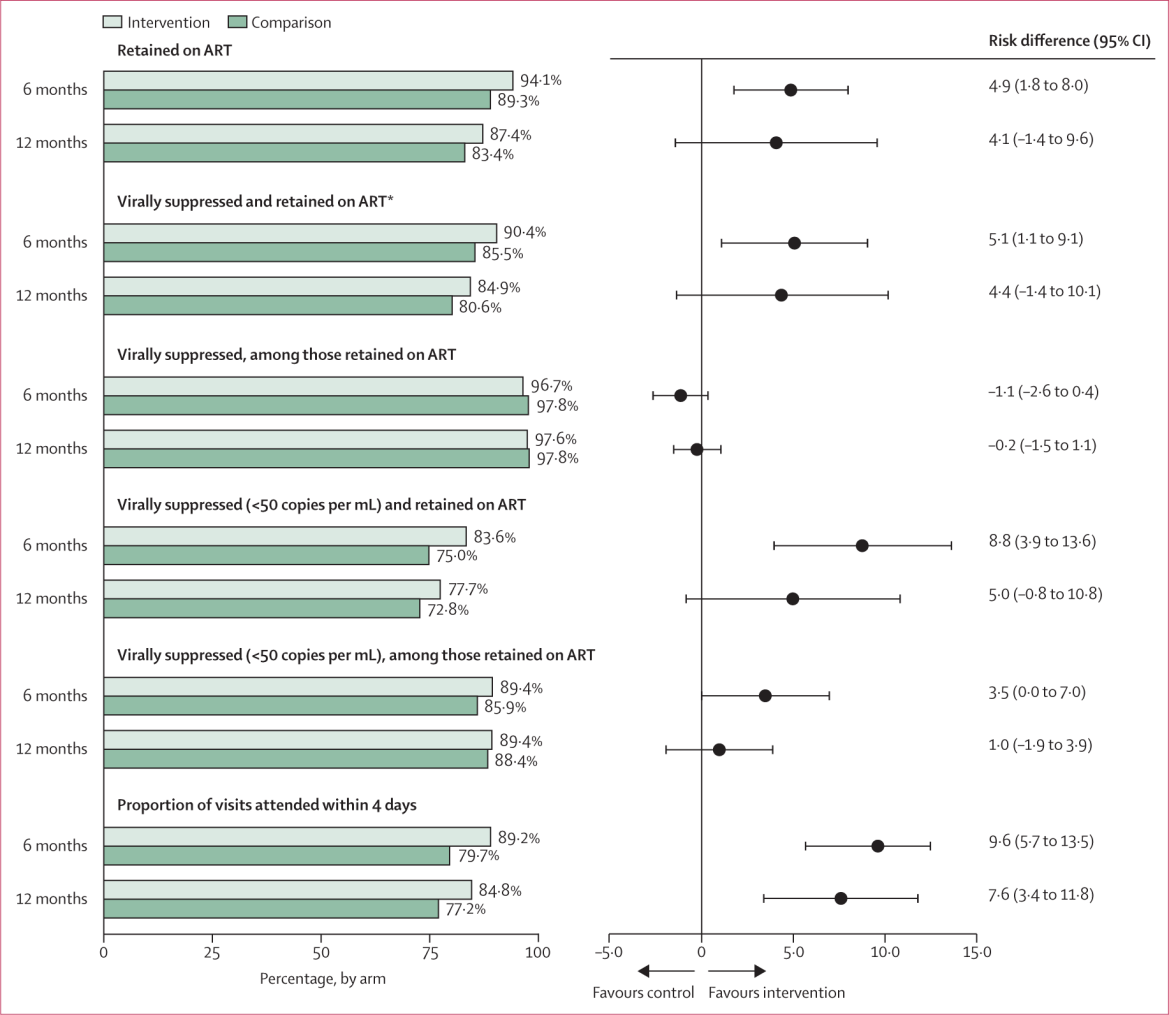
Effects of financial incentives on viral suppression and retention on ART at 6 months and 12 months, Tanzania 2021–23 *Prespecified primary outcome; risk differences are adjusted for region (model 1 estimates) and expressed as percentage point differences.

Overall, 1536 participants (88%) at 6 months and 1575 (83%) at 12 months were on ART with viral suppression (<1000 copies per mL). At 12 months, 6 months after the intervention had ended, 709 (81%) participants in control facilities were on ART with viral suppression (primary outcome), compared with 866 (85%) in the intervention group (risk difference [RD] 4.4 percentage points, 95% CI –1.4 to 10.1; permutation test p=0.35); this was not statistically different from the effect at 6 months (p=0.69). These findings were driven primarily through improved retention on ART at intervention facilities (RD 4.9 percentage points, 95% CI 1.8 to 8.0 at 6 months; RD 4.1 percentage points, 95% CI −1.4 to 9.6 at 12 months). At 6 months, 678 (86%) participants in control facilities were on ART with viral suppression, compared with 858 (90%) in the intervention group (adjusted RD [aRD] 5.1 percentage points, 95% CI 1.1 to 9.1; permutation test p=0.06; fig 2). Among those who were retained on ART, the proportion with viral suppression at 6 months and 12 months was high (roughly 98%) in both the intervention and control group. Results were consistent across various model specifications (table 2).

1388 (80%) and 1434 (75%) participants were retained on ART with viral suppression at the lower threshold of less than 50 copies per mL at 6 months and 12 months, respectively. At 6 months, the intervention was nearly twice as effective at achieving this lower level of viral suppression than the higher threshold (RD 8.8 percentage points, 95% CI 3.9 to 13.6). 6 months after the intervention ended, the effect was smaller and no longer significant (5.0 percentage points, −0.8 to 10.8 at 12 months); however, this effect was not statistically different from the 6-month effect (p=0.10; figure 2).

Patients at intervention facilities had a significantly higher proportion of scheduled visits attended on time (within 4 days) compared with those at control facilities, both at 6 months (RD 9.6 percentage points, 95% CI 5.7 to 13.5) and 12 months (7.6 percentage points, 3.4 to 11.8) months (figure 2). We hypothesised that this finding could partly explain the larger impacts on the lower threshold for defining viral suppression, which could require stricter daily adherence than necessary for the higher threshold of 1000 copies per mL. To test this possible pathway of impact, we used ART dispensing and visit records to explore differences in the number of days of missed ART (ie, days not in possession of ART) and the number of days late to visits among those retained on ART at 6 months and 12 months. Participants retained on ART at 6 months at intervention sites missed 1.4 (95% CI −0.1 to 2.9) fewer days of ART and were 5.6 (3.7 to 7.5) fewer cumulative days late to scheduled visits during the first 6 months of the study compared with participants at control sites. Among those retained on ART at 12 months, these differences were slightly larger, with 1.8 (−1.9 to 5.4) fewer missed days of ART and 6.6 (1.9 to 11.3) fewer cumulative days late to scheduled visits strengthening evidence for our hypothesis.

Throughout the duration of the intervention, 5342 cash transfers were sent to 1025 (97%) of 1059 intervention participants within 48 h of appointment attendance (a timely disbursement). On average, each participant received 5.2 (range 4.5 to 5.6) timely cash transfers. Clinic-level fidelity to the intervention was extremely high across all 16 intervention sites (range 0.89 to 0.98, where 1 indicates perfect implementation of the intervention), and thus the analysis of the primary outcome that accounted for clinic-level fidelity to the intervention was not dramatically different from the ITT results (RD 5.4 percentage points, 95% CI 1.2 to 9.5 at 6 month; 4.6 percentage points, −1.5 to 11.0 at 12 months; table 2).

**Figure 3:**
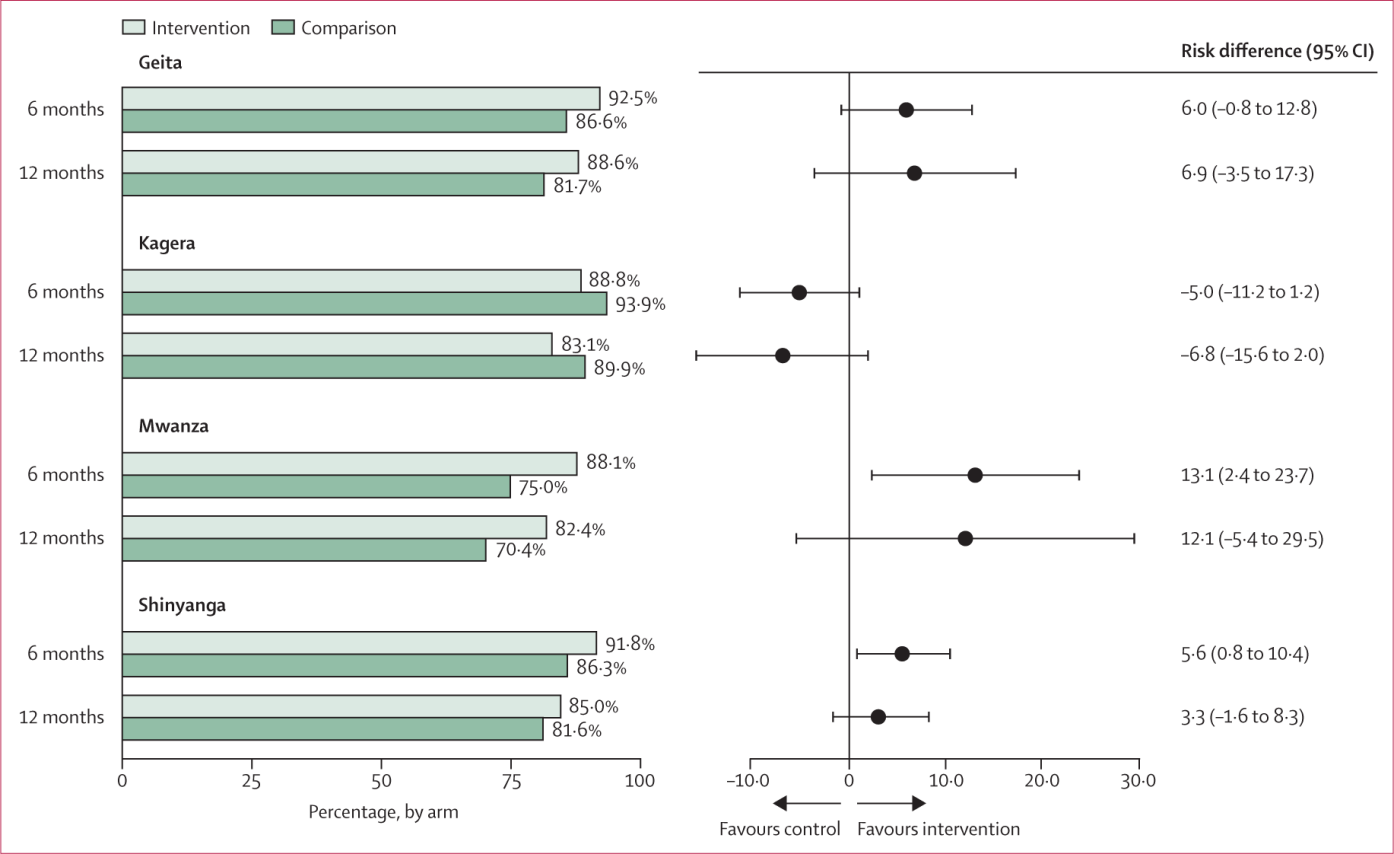
Effects of financial incentives on viral suppression with retention on ART (primary outcome), by region, Tanzania 2021–23 Risk differences are adjusted for region (model 1 estimates) and expressed as percentage point differences.

Per our prespecified analysis plan, we also evaluated ITT effects on primary outcomes for subgroups defined by region, facility type, gender, age, and household wealth. We found substantial geographic heterogeneity in the ITT analysis of viral suppression with retention on ART at both 6 months and 12 months (figure 3). ITT effects among participants in Geita and Shinyanga regions did not differ substantially from the overall results; however, effects of the intervention were much larger among participants in Mwanza and substantially lower (favouring control) in Kagera. No heterogeneity in intervention effects were observed by facility type, gender, age, or household wealth (table 3).

Due to substantially different intervention effects in Kagera compared with the other three regions (interaction p=0.014), we ran our primary models on a subsample excluding the Kagera facilities. We found larger and significant effects of the intervention at both 6 months and 12 months for retention on ART and viral suppression at the threshold of less than 1000 copies per mL (RD 8.0 percentage points, 95% CI 3.6 to 12.0 at 6 month; 7.4 percentage points, 0.6 to 14.0 at 12 months), as well as for retention on ART with viral suppression using the lower threshold of less than 50 copies per mL (RD 10.0 percentage points, 95% CI 4.6 to 16.0 at 6 months; 8.4 percentage points, 2.1 to 15.0 at 12 months; appendix 2 p 1). These effects were similar to the results that excluded only the two Kagera intervention facilities with a greater proportion of migrant workers (appendix 2 p 2).

Given that more than 5% of the sample was missing the primary outcome at 6 months, we reran our analysis with missing viral suppression status imputed via multiple imputation per our prespecified analysis plan. The effects of the intervention on the primary outcome at 6 months and 12 months were consistent with the complete case analysis (pooled RD 5.3 percentage points, 95% CI 1.5 to 9.1 at 6 months; 4.5 percentage points, −1.1 to 10.0 at 12 months; appendix 2 p 3).

Adverse events included 106 deaths (56 in the control group and 50 in the intervention group), none related to study participation.

## Discussion

This hybrid effectiveness–implementation trial is one of the largest and most rigorous studies to date of the short-term and longer-term effects of small, short-term financial incentives on viral suppression among people starting HIV treatment, a period often characterised by high mortality and attrition from care.^22^ The intervention was theoretically based; benefitted from the input and advice of local experts, stakeholders, and the Tanzania Ministry of Health; and was previously evaluated and refined in two randomised pilot studies, in which it showed safety and effect on ART possession^13^ and short-term viral suppression.^17^ In this study, which expanded the intervention to 32 clinics in four regions, we found that retention on ART with viral suppression is high in the era of dolutegravir-based ART regimens.^23^ In this setting of potent ART, financial incentives for visit attendance yielded modest but significantly improved levels of viral suppression at 6 months when using the prespecified less than 1000 copies per mL cutoff for suppression; this effect was statistically similar but no longer significant at 12 months (primary outcome), 6 months after discontinuation of incentives. However, reanalysis with a modern threshold for virological success of less than 50 copies per mL revealed stronger effects of the intervention, possibly related to small improvements in compliance with visit schedules or daily ART adherence, which are both crucial for achieving very low viral loads, especially in the context of dolutegravir-based regimens.^24^ Taken together, these findings are consistent with some, but not all, studies,^10,17,25,26^ showing safety and modest benefits of short-term financial incentives for improving HIV viral suppression, and highlight the need to better understand which subgroups would most benefit from incentives to support engagement in HIV care.

The level of viral suppression in the control group was high and is a testament to Tanzania’s concerted efforts to achieve the 95–95-95 goals and scale-up dolutegravir-based regimens.^27^ We observed at least a 10 percentage point positive difference in viral suppression at 6 months after ART initiation in the control group of the current study versus the pilot randomised trial, which were approximately 3 years apart.^17^ Against this backdrop, where previous studies have found that most people achieve viral suppression,^28,29^ some outcomes approached the success ceiling, such as 98% of those retained on ART having viral suppression. We also cannot rule out a possible small benefit of the control group’s receipt of the mHealth system to monitor visits and send SMS visit reminders. Regardless, there remains a subgroup of people living with HIV who need better access or support; for example, one in ten people in the control group were no longer on ART at 6 months. We are currently evaluating the use of machine learning paired with routine HIV care data to predict people living with HIV at high risk of disengagement from care and offer support through a combination intervention of financial incentives and empathy-based, person-centered counselling (ClinicalTrials.gov NCT05373095).

Our examination of both the immediate and longer-term effects of the intervention after its discontinuation, which has been unresolved in the literature, is a major contribution of this study. Qualitative and quantitative research by our team^13,30,31^ did not find evidence to support the hypothesis that incentivised people living with HIV could have worse care engagement than their non-incentivised counterparts after incentives are discontinued. The CI for the risk difference we observed at 12 months (−1.4 to 9.6), 6 months after the incentives ended, rules out the possibility of any meaningful adverse effect. Moreover, timely visit attendance, which was directly incentivised by the intervention, was significantly higher in intervention sites 6 months after their discontinuation.

However, whether the incentives as implemented in our study have longer-term benefits after their discontinuation is less clear. Compared with the significant effect observed at 6 months, the effect size at 12 months (primary outcome) was slightly smaller (although not statistically different) but the SE was larger; therefore we cannot conclude that the benefit we observe at 12 months is statistically different from zero. However, the CI is also not sufficiently precise to rule out the possibility of modest long-term benefits. In a previous long-term study of pilot study participants, we found small (but not statistically significant) possible benefits^13,30^ of incentives after their discontinuation. Nevertheless, it might be unreasonable to expect that incentives would have substantial benefits after their removal, as is the case with most non-vaccine biomedical interventions after their discontinuation. Taken together, the results from this study confirm that short-term incentives do no long-term harm to intrinsic motivation, confirming their suitability for specific subgroups or windows of opportunity, like HIV treatment initiation, when the risk of disengaging from care is high and the chances of habit formation are strongest.

There was substantial effect heterogeneity across the four regions. We anticipated possible regional differences in effectiveness that could be due to variability in socioeconomic status between regions, which was one of several factors that led to selection of the higher incentive amount for this trial.^17,18^ However, we observed null findings in one region compared with strong and consistent benefits of the intervention in the other three; a result that was also inconsistent with the two pilot trials. A post-hoc investigation revealed that two health facilities in the region had substantially higher rates of ART discontinuation than elsewhere in the study, possibly related to their serving of a large proportion of migrant workers. Both facilities were randomly assigned to the intervention group. When restricted to the other three regions, the effectiveness of the intervention was larger and closer to the result observed in the pilot study.^17^

This study benefited from a multiphase process to carefully tailor the intervention and implementation strategy to the local context and the needs of potential beneficiaries. This resulted in an implementation model that relied on a mHealth system with biometric identification, automatic delivery of cash transfers via mobile money, SMS distribution of visit reminders, and automatic importing of viral load results.^16^ This mHealth system, designed by a Tanzanian technology firm, enabled nearly real-time tracking of patient visits (all sites) and cash distribution (intervention sites), which has been a practical challenge in other studies of cash transfers.^32^ This study shows that incentive programmes can indeed be securely brought to scale through use of an easy-to-use digital platform at HIV primary care clinics.

Our intervention was designed with local stakeholders to pair with Tanzania’s clinical management plan which calls for monthly visits during the first 6 months of ART followed by an eligibility assessment for multimonth dispensing for subsequent visits.^33^ Whether results would be similar had the study been done with ART-experienced individuals with visits scheduled every 60, 90, or 180 days is unknown, nor is the applicability of these findings to the future promise of long-acting, injectable ART. However, challenges with retention in care and adherence to ART will remain for a subset of vulnerable people living with HIV even with long-acting ART. Behavioural science approaches, including but not limited to financial incentives, remain an important component of a comprehensive, equity-based strategy to ensure that efficacious biomedical tools translate to population-level effectiveness among diverse groups.^11^

This study used a rigorous, effectiveness–implementation design and is the largest study to date of the effectiveness of financial incentives on HIV viral suppression in a low-income or middle-income country;^10^ only HTPN 065 conducted in the USA is larger.^25^ We used best practices for data collection and participant tracing to minimise or eliminate bias from silent transfers. Missing data were minimised and the findings were robust to several model specifications, including models using multiple imputation to account for missing outcome data. Nevertheless, the study has important limitations. The study was designed and conducted over an extended timeline due to COVID-19 delays, which could introduce background temporal changes. One such change was the roll-out of dolutegravir-based regimens in Tanzania in 2019–20, which made the results less comparable with previous studies, including the pilot studies done between 2013–15 and 2018–19.^27^ In addition, contemporaneous changes in the viral load thresholds for defining success on ART occurring during the trial meant that some outcomes relevant to clinical practice and policies were not prespecified.

In conclusion, in this large effectiveness–implementation trial in Tanzania, we found that retention on ART with viral suppression was high. In this setting, financial incentives yielded modest but considerably improved outcomes at 6 months, with similar, small benefits likely to be retained at 12 months, 6 months after incentives were discontinued. When using a stricter, but increasingly common threshold for viral suppression, the short-term effect of incentives was larger which could warrant their incorporation into comprehensive programs to support people initiating or re-initiating ART. In addition, these findings also suggest the need to understand subgroups who would most benefit from incentives to support engagement in HIV care.

## Supplementary Material

Appendix 2

Appendix 1

## Figures and Tables

**Table 1:** Baseline characteristics of intent-to-treat sample, Tanzania 2021–22

	Full sample (32 clinics; 1990 participants)	Intervention (16 clinics; 1059 participants)	Control (16 clinics; 931 participants)

**Clinic characteristics**			
Region			
Geita	8 (25%)	4 (25%)	4 (25%)
Kagera	8 (25%)	4 (25%)	4 (25%)
Mwanza	8 (25%)	4 (25%)	4 (25%)
Shinyanga	8 (25%)	4 (25%)	4 (25%)
Facility type			
Dispensary	9 (28%)	4 (25%)	5 (31%)
Health Center	13 (41%)	5 (31%)	8 (50%)
Hospital	10 (31%)	7 (44%)	3 (19%)
Log average ART initiates per quarter in 2019	4.89 (0.47)	5.03 (0.40)	4.75 (0.50)
Distance to a major city (km)	46.00 (27.53)	44.56 (28.61)	47.44 (27.26)
Proximity (<5 km) to a major road	16 (50%)	9 (56%)	7 (44%)
**Participant characteristics**			
Age, years	36.70 (11.55)	36.46 (11.48)	36.98 (11.62)
Female	1185 (59.5%)	652 (61.6%)	533 (57.3%)
Male	805 (40.5%)	407 (38.4%)	398 (42.7%)
Married or partnered*	1193 (60.7%)	624 (59.4%)	569 (62.1%)
Head of household*	1110 (56.5%)	581 (55.3%)	529 (57.8%)
Language*			
Swahili	700 (35.6%)	384 (36.6%)	316 (34.5%)
Sukuma	957 (48.7%)	492 (46.9%)	465 (50.8%)
Haya	241 (12.3%)	129 (12.3%)	112 (12.2%)
Other	68 (3.5%)	45 (4.3%)	23 (2.5%)
Educational attainment†			
No formal education	494 (25.2%)	268 (25.5%)	226 (24.8%)
Some primary	333 (17.0%)	184 (17.5%)	149 (16.4%)
Completed primary	899 (45.9%)	482 (45.9%)	417 (45.9%)
More than primary	231 (11.8%)	115 (11.0%)	116 (12.7%)
Worked in the past 7 days*	1231 (62.6%)	683 (65.0%)	548 (59.8%)
Household size, member‡	4.85 (3.20)	4.71 (3.03)	5.02 (3.38)
Moderate or severe household hunger (HHS-3)§	140 (7.1%)	81 (7.7%)	59 (6.4%)
Wealth index	6.74 (8.89)	6.07 (7.34)	7.51 (10.32)
Depression (PHQ-2)*	1317 (67.0%)	682 (65.0%)	635 (69.3%)
Anxiety (GAD-2)*	1201 (61.1%)	627 (59.7%)	574 (62.7%)
Days on ART at enrolment	3.50 (6.64)	3.56 (6.53)	3.42 (6.77)
Dolutegravir-based regimen¶	1973 (99.8%)	1054 (100%)	919 (99.7%)
Weight, kg||	56.93 (10.07)	56.84 (9.80)	57.04 (10.38)
HIV WHO clinical stage			
Stage 1	1348 (68.7%)	703 (67.1%)	645 (70.4%)
Stage 2	404 (20.6%)	206 (19.7%)	198 (21.6%)
Stage 3	189 (9.6%)	123 (11.7%)	66 (7.2%)
Stage 4	22 (1.1%)	15 (1.4%)	7 (0.8%)
Missing	27 (1.4%)	12 (1.1%)	15 (1.6%)
Pregnant**	52 (4.6%)	20 (3.2%)	32 (6.2%)

Data are n (%) or mean (SD). Missing data were excluded from denominators:

* n=24,

‡ n=30,

† n=114,

§ n=11,

¶ n=14,

|| n=36, and

** n=56 (among 1185 females). ART=antiretroviral therapy. HHS=Household Hunger Scale. PHQ=Patient Health Questionnaire. GAD=generalised anxiety disorder.

**Table 2:** Effects of financial incentives and intervention fidelity on retention on ART and viral suppression at 6 months and 12 months, Tanzania 2021–23

	N	Financial incentives group (proportion)	Control group (proportion)	Intent-to-treat analysis (risk difference [95% CI])			Fidelity analysis* (risk difference [95% CI])
				Model 1	Model 2	Model 3	Model 1

**6 months**							
Retained on ART	1990	0.941	0.893	0.049 (0.018 to 0.080)	0.044 (0.009 to 0.078)	0.048 (0.012 to 0.084)	0.051 (0.018 to 0.084)
Virally suppressed (<1000 copies per mL) and retained on ART	1742	0.904	0.855	0.051 (0.011 to 0.091)	0.051 (0.009 to 0.093)	0.056 (0.012 to 0.100)	0.054 (0.012 to 0.095)
Virally suppressed (<1000 copies per mL), among those retained on ART	1580	0.967	0.978	−0.011 (−0.026 to 0.004)	−0.006 (−0.020 to 0.009)	−0.006 (−0.020 to 0.009)	−0.011 (−0.027 to 0.004)
Virally suppressed (<50 copies per mL) and retained on ART	1742	0.836	0.750	0.088 (0.039 to 0.136)	0.097 (0.049 to 0.145)	0.099 (0.049 to 0.148)	0.092 (0.041 to 0.140)
Virally suppressed (<50 copies per mL), among those retained on ART	1580	0.894	0.859	0.035 (0.000 to 0.070)	0.051 (0.021 to 0.081)	0.046 (0.017 to 0.075)	0.037 (0.000 to 0.074)
Proportion of visits attended on time	1990	0.892	0.797	0.096 (0.057 to 0.135)	0.085 (0.045 to 0.126)	0.089 (0.045 to 0.132)	0.100 (0.060 to 0.140)
**12 months**							
Retained on ART	1990	0.874	0.834	0.041 (−0.014 to 0.096)	0.028 (−0.024 to 0.079)	0.033 (−0.019 to 0.086)	0.043 (−0.015 to 0.100)
Virally suppressed (<1000 copies per mL) and retained on ART†	1900	0.849	0.806	0.044 (−0.014 to 0.101)	0.034 (−0.022 to 0.089)	0.041 (−0.016 to 0.098)	0.046 (−0.015 to 0.110)
Virally suppressed (<1000 copies per mL), among those retained on ART	1612	0.976	0.978	−0.002 (−0.015 to 0.011)	0.001 (−0.012 to 0.013)	0.002 (−0.010 to 0.014)	−0.002 (−0.015 to 0.011)
Virally suppressed (<50 copies per mL) and retained on ART	1900	0.777	0.728	0.050 (−0.008 to 0.108)	0.036 (−0.020 to 0.093)	0.040 (−0.020 to 0.101)	0.052 (−0.009 to 0.110)
Virally suppressed (<50 copies per mL), among those retained on ART	1612	0.894	0.884	0.010 (−0.019 to 0.039)	0.008 (−0.019 to 0.034)	0.006 (−0.023 to 0.035)	0.011 (−0.020 to 0.041)
Proportion of visits attended on time	1990	0.848	0.772	0.076 (0.034 to 0.118)	0.061 (0.020 to 0.103)	0.064 (0.020 to 0.108)	0.080 (0.036 to 0.120)

Data are linear probability model estimates of adjusted risk differences and 95% CIs with robust SEs clustered by clinic. Model 1 (primary model) included region fixed effects to account for the stratified study design. Model 2 adjusted for region plus clinic-level covariates, including facility type, log average ART initiates per quarter in 2019, distance to a major city, and proximity (<5 km) to a major road. Model 3 included all covariates in model 2 plus participanťs age, gender, and WHO clinical stage. Model 1 fidelity analysis included region fixed effects covariate. Participants on ART but missing viral loads at 6 months (n=248) and 12 months (n=90) were excluded from the analysis of viral suppression among those on ART. ART=antiretroviral therapy.

* Fidelity analysis RDs were estimated with a two-stage least-squares instrumental variable model to account for clinic-level differences in implementation fidelity.

† Prespecified primary outcome.

**Table 3:** Effects of financial incentives on retention on ART with viral suppression (primary outcome) within subgroups based on clinic and participant characteristics, Tanzania 2021–23

	N	Financial incentives group (proportion)	Control group (proportion)	Intent-to-treat analysis (risk difference [95% CI])

**Region**				
Geita				
6 months	456	0.925	0.866	0.060 (−0.008 to 0.128)
12 months	513	0.886	0.817	0.069 (−0.035 to 0.173)
Kagera				
6 months	385	0.888	0.939	−0.050 (−0.112 to 0.012)
12 months	397	0.831	0.899	−0.068 (−0.156 to 0.020)
Mwanza				
6 months	423	0.881	0.750	0.131 (0.024 to 0.237)
12 months	472	0.824	0.704	0.121 (−0.054 to 0.295)
Shinyanga				
6 months	478	0.918	0.863	0.056 (0.008 to 0.104)
12 months	518	0.850	0.816	0.033 (−0.016 to 0.083)
**Facility type**				
Dispensary				
6 months	477	0.926	0.905	0.022 (−0.042 to 0.085)
12 months	500	0.906	0.863	0.044 (−0.034 to 0.121)
Health Center				
6 months	725	0.913	0.831	0.082 (0.014 to 0.150)
12 months	813	0.810	0.781	0.030 (−0.062 to 0.121)
Hospital				
6 months	540	0.882	0.849	0.033 (−0.092 to 0.157)
12 months	587	0.844	0.797	0.047 (−0.117 to 0.211)
**Gender**				
Male				
6 months	719	0.886	0.852	0.045 (−0.012 to 0.102)
12 months	768	0.853	0.807	0.056 (−0.017 to 0.130)
Female				
6 months	1023	0.916	0.857	0.059 (0.017 to 0.100)
12 months	1132	0.846	0.805	0.038 (−0.023 to 0.098)
**Age, years**				
18–24				
6 months	252	0.915	0.838	0.079 (−0.018 to 0.177)
12 months	283	0.841	0.815	0.018 (−0.084 to 0.120)
25–34				
6 months	601	0.882	0.864	0.019 (−0.049 to 0.087)
12 months	657	0.846	0.814	0.031 (−0.051 to 0.113)
≥35				
6 months	889	0.916	0.853	0.070 (0.023 to 0.117)
12 months	960	0.853	0.797	0.061 (−0.007 to 0.130)
**Wealth index**				
Low				
6 months	590	0.903	0.845	0.057 (−0.007 to 0.122)
12 months	637	0.881	0.798	0.081 (−0.003 to 0.165)
Medium				
6 months	568	0.896	0.831	0.075 (0.010 to 0.140)
12 months	630	0.837	0.787	0.053 (−0.021 to 0.127)
High				
6 months	583	0.914	0.884	0.033 (−0.012 to 0.077)
12 months	632	0.824	0.828	−0.004 (−0.071 to 0.064)

Data are linear probability model estimates of adjusted risk differences and 95% CIs with robust SEs clustered by clinic. Models used for subgroup analyses by gender, age, and household wealth include region fixed effects to account for the stratified study design.

## Data Availability

Individual de-identified participant data that underlie the results reported in this Article and R code used to generate the results in this study are available upon request from the authors after receipt of a methodologically sound proposal from interested researchers. We will also make available the study protocol and statistical analysis plan. Data will be available beginning 3 months and ending 5 years following Article publication. Proposals should be directed to smccoy@berkeley.edu; to gain access, data requestors will need to sign a data access agreement and show evidence that the proposed use of the data has been approved by an independent ethical review committee identified for this purpose.

## Research in context

### Evidence before this study

Previous studies have found that financial and non-financial incentives can increase HIV testing, retention in HIV care, and adherence to antiretroviral therapy (ART). However, a PubMed search reveals that evidence about the effectiveness of incentives for improving viral suppression has been scarce, especially in sub-Saharan Africa (search string: “HIV” AND “incentives” AND “viral suppression” AND (“evaluation” OR “effectiveness” OR “trial” OR “randomized” OR “experiment” [tiab])). For example, the meta-analysis of ten mostly small studies (<500 participants) by Krishnamoorthy and colleagues found that the pooled relative risk for the relationship between financial incentives and HIV viral suppression was 1.08, with less than half of the studies having been conducted in Africa. Some have hypothesised that incentives might harm intrinsic motivation for daily ART adherence, resulting in worse health outcomes after incentives are discontinued, although no studies have formally examined this possibility. In addition, few studies have studied real-world implementation models for incentive interventions that could be widely scaled.

### Added value of this study

This hybrid effectiveness–implementation trial is the largest and most rigorous study to date of the short-term and longer-term effects of small, short-term financial incentives on viral suppression among people starting HIV treatment in sub-Saharan Africa. We found that financial incentives yielded modest but significantly improved levels of viral suppression at 6 months; this effect was statistically similar at 12 months, 6 months after discontinuation of incentives. Incentives did not harm retention or viral suppression at 12 months. The effect of incentives was stronger when using a stricter threshold for virological suppression, which was possibly related to small improvements in compliance with visit schedules or daily adherence, or both, that might have otherwise been unobserved when using a higher threshold for viral suppression. The findings were robust to several model specifications and exploratory analyses. In addition, a customised mobile health technology (mHealth) system with biometric identification enabled staff across 16 intervention clinics to seamlessly send incentives via mobile money while experiencing added benefits like alerts about virological monitoring and SMS visit reminders.

### Implications of all the available evidence

The available evidence suggests that when appropriately tailored to the desired behaviour and context, financial incentives can improve the proportion of people with HIV viral suppression, with no longer-term harms. This specific study demonstrates the potential value of a financial incentive programme delivered through a scalable mHealth implementation model to increase the use of HIV services and achieve viral suppression, a crucial goal for ending the HIV epidemic. Future research may focus on which subgroups would most benefit from incentives to support engagement in HIV care.
