# Supplementary material for: Impact of financial incentives on viral suppression among adults initiating HIV treatment in Tanzania: a hybrid effectiveness–implementation trial
Source: Lancet HIV. Author manuscript; Available in PMC 2024 Oct 7. (PMC11457171; doi:10.1016/S2352-3018(24)00149-8)
Supplement: Appendix 2 [file NIHMS2021906-supplement-Appendix_2.pdf]

THE LANCET
HIV

Supplementary appendix 2

This appendix formed part of the original submission and has been peer reviewed. We post it as supplied by the authors.

Supplement to: Njau PF, Katabaro E, Winters S, et al. Impact of financial incentives on viral suppression among adults initiating HIV treatment in Tanzania: a hybrid effectiveness–implementation trial. *Lancet HIV* 2024; published online Aug 1. https://doi.org/10.1016/S2352-3018(24)00149-8.

Supplementary Table 1. Effects of financial incentives on retention on ART and viral suppression at 6 and 12 months, excluding all Kagera facilities, Tanzania 2021-2023

	**N**	**Financial incentives proportion**	**Comparison proportion**	**Intent-to-treat analysis Risk Difference (95% CI)**
**Six months**				
Retained on ART	1577	0.941	0.875	**0.067 (0.033,0.100)**
Virally suppressed (<1000 cp/mL) and retained on ART	1357	0.908	0.831	**0.080 (0.036,0.120)**
Virally suppressed (<1000 cp/mL), among those retained on ART	1215	0.973	0.979	-0.005 (-0.023,0.012)
Virally suppressed (<50 cp/mL) and retained on ART	1357	0.843	0.743	**0.103 (0.046,0.160)**
Virally suppressed (<50 cp/mL), among those retained on ART	1215	0.902	0.875	0.029 (-0.012,0.069)
Proportion of visits attended on time	1577	0.898	0.792	**0.113 (0.068,0.158)**
**Twelve months**				
Retained on ART	1577	0.879	0.805	**0.074 (0.014,0.130)**
Virally suppressed (<1000 cp/mL) and retained on ART*	1503	0.854	0.782	**0.073 (0.006,0.140)**
Virally suppressed (<1000 cp/mL), among those retained on ART	1257	0.977	0.986	-0.008 (-0.021,0.004)
Virally suppressed (<50 cp/mL) and retained on ART	1503	0.788	0.705	**0.084 (0.021,0.150)**
Virally suppressed (<50 cp/mL), among those retained on ART	1257	0.901	0.889	0.014 (-0.018,0.046)
Proportion of visits attended on time	1577	0.859	0.767	**0.098 (0.051,0.145)**

Supplementary Table 2. Effects of financial incentives on retention on ART and viral suppression at 6 and 12 months, excluding two Kagera facilities with a higher proportion of migrant workers, Tanzania 2021-2023

	**N**	**Financial incentives proportion**	**Comparison proportion**	**Intent-to-treat analysis Risk Difference (95% CI)**
**Six months**				
Retained on ART	1903	0.945	0.893	**0.057 (0.027,0.087)**
Virally suppressed (<1000 cp/mL) and retained on ART	1668	0.911	0.855	**0.063 (0.024,0.101)**
Virally suppressed (<1000 cp/mL), among those retained on ART	1515	0.970	0.978	-0.009 (-0.024,0.006)
Virally suppressed (<50 cp/mL) and retained on ART	1668	0.846	0.750	**0.100 (0.053,0.147)**
Virally suppressed (<50 cp/mL), among those retained on ART	1515	0.900	0.859	**0.039 (0.005,0.074)**
Proportion of visits attended on time	1903	0.894	0.797	**0.098 (0.057,0.138)**
**Twelve months**				
Retained on ART	1903	0.884	0.834	**0.056 (0.003,0.109)**
Virally suppressed (<1000 cp/mL) and retained on ART*	1818	0.858	0.806	**0.058 (0.001,0.114)**
Virally suppressed (<1000 cp/mL), among those retained on ART	1550	0.976	0.978	-0.004 (-0.016,0.009)
Virally suppressed (<50 cp/mL) and retained on ART	1818	0.785	0.728	**0.060 (0.003,0.118)**
Virally suppressed (<50 cp/mL), among those retained on ART	1550	0.892	0.884	0.006 (-0.023,0.035)
Proportion of visits attended on time	1903	0.850	0.772	**0.077 (0.033,0.121)**

Supplementary Table 3. Effects of financial incentives on retention on ART with viral suppression (primary outcome), using multiple imputation, Tanzania 2021-2023

	N	Financial incentives proportion	Comparison proportion	Intent-to-treat analysis Pooled Risk Difference (95% CI) (1)	(2)	(3)
**Six months**						
Virally suppressed (<1000 cp/mL) and retained on ART	1990	0.903	0.850	**0.053 (0.015,0.091)**	**0.052 (0.013,0.091)**	**0.056 (0.016,0.097)**
**Twelve months**						
Virally suppressed (<1000 cp/mL) and retained on ART	1990	0.849	0.804	0.045 (-0.011,0.100)	0.035 (-0.018,0.088)	0.042 (-0.012,0.097)
Region FE				*X*	*X*	*X*
Facility level					*X*	*X*
log ART initiates					*X*	*X*
Proximity to major road					*X*	*X*
Distance to major city					*X*	*X*
Age, years						*X*
Gender						*X*
WHO clinical stage						*X*

Linear probability models were used to estimate adjusted risk differences (RD) and 95% confidence intervals (CI), with robust standard errors clustered by clinic, for 20 multiply imputed datasets. Pooled estimates are presented here. RDs significant at the alpha=0.05 level are in bold. Covariates included in the models are marked with an X.
