# Supplementary material for: Impact of financial incentives on viral suppression among adults initiating HIV treatment in Tanzania: a hybrid effectiveness–implementation trial
Source: Lancet HIV. Author manuscript; Available in PMC 2024 Oct 7. (PMC11457171; doi:10.1016/S2352-3018(24)00149-8)
Supplement: Appendix 1 [file NIHMS2021906-supplement-Appendix_1.pdf]

THE LANCET
HIV

Supplementary appendix 1

This translation in Swahili was submitted by the authors and we reproduce it as supplied. It has not been peer reviewed. *The Lancet*'s editorial processes have only been applied to the original in English, which should serve as reference for this manuscript.

Tafsiri hii katika Swahili iliwasilishwa na waandishi na tunatengeneza tena kama hutolewa. Haijapitiwa. Mchakato wa hariri wa Lancet Global Health umetumika tu kwa asili kwa Kiingereza, ambayo inapaswa kutumika kama kumbukumbu kwa muswada hii.

Supplement to: Njau PF, Katabaro E, Winters S, et al. Impact of financial incentives on viral suppression among adults initiating HIV treatment in Tanzania: a hybrid effectiveness–implementation trial. *Lancet HIV* 2024; published online Aug 1. https://doi.org/10.1016/S2352-3018(24)00149-8.

Muhtasari

Usuli

Vichocheo vidogo vinaweza kuboresha ushiriki kwenye huduma za VVU. Tulitathmini athari za muda mfupi na mrefu za vichocheo vya kifedha kwa mahudhurio kwenye ukandamizaji wa virusi kwa watu wazima wanaoanza tiba ya kupambana na virusi (ART) nchini Tanzania.

Mbinu

Katika utafiti wa Aina ya 1 ya mseto wa ufanisi na utekelezaji, tulichagua kwa nasibu (1:1) kliniki 32 za huduma za msingi za VVU katika mikoa minne kwa huduma za kawaida (kundi linganifu) au afua (huduma za kawaida pamoja na magawio ya fedha yasiyozidi 6 kila mwezi [Tsh. 22,500/=, karibu USD 10, kila moja], yanayotegemeana na kuhudhuria miadi). Watu wazima (wenye umri wa miaka 18 au zaidi) wanaoanza ART (chini ya siku 30) walio na simu ya mkononi na wasio na mipango ya kuhamia kituo kingine walistahiki. Matokeo ya msingi (primary outcomes) yalikuwa ni kubakia kwenye ART na udhibiti wa virusi (chini ya nakala 1000 kwa mL) kwa miezi 12. Matokeo ya ziada (secondary outcomes) yalihusisha kubakia kwenye ART na ufubazaji wa virusi kwenye miezi 6 na ufubazaji wa virusi kwenye miezi 6 na 12 kwa kutumia kiwango cha chini cha kupima na kugundua mzigo wa virusi (chini ya nakala 50 kwa mL). Uchambuzi wa nia ya kutibu na jaribio maboresho la kulingana na makundi (intent-to-treat analysis and a cluster-based permutation test) vilitumiwa kutathmini athari za motisha za kifedha kwenye matokeo. Jaribio hili limesajiliwa na ClinicalTrials.gov, NCT04201353, na limekamilika.

Matokeo

Kati ya Mei 28, 2021 na Machi 8, 2022, washiriki 1990 (wanaume 805 na wanawake 1185) walisajiliwa kwenye utafiti. Washiriki 1059 walipangwa kwenye kundi la afua, washiriki 931 walipangwa kwenye kundi linganifu. Kwa ujumla, washiriki 1536 (88%) kwenye miezi 6 na 1575 (83%) kwenye miezi 12 walikuwa kwenye ART na walikuwa wamefubaza. Kwenye miezi 12, miezi 6 baada ya afua kumalizika, washiriki 866 (85%) wa afua kulinganisha na washiriki 709 (81%) kwenye kundi linganifu walikuwa na wingi wa virusi wa chini ya nakala 1000 (aRD=4·4 pointi za asilimia (percentage points), 95% CI: -1·4, 10·1). Kwenye miezi 6, washiriki 858 (90%) wa afua walikuwa kwenye ART na wakiwa wamefubaza virusi kwa chini ya nakala 1000 ikilinganishwa na 678 (86%) wa kwenye kundi linganifu (aRD=5·1 pointi za asilimia (percentage points), 95% CI 1·1 hadi 9·1, jaribio la mzunguko (permutation test), p=0.06). Athari zilikuwa kubwa zaidi kwa miezi 6 na 12 kwa kutumia kiwango cha chini cha kupima na kugundua ufubazaji virusi, na kulikuwa na athari kubwa zenye maana kitafiti zilizotofautiana kimkoa. Matukio mabaya yalijumuisha vifo 106 (56 katika kundi inganifu na 50 katika kundi la afua), hakuna hata moja lililohusiana na ushiriki wa utafiti.

Tafsiri

Motisha za muda mfupi kwa ajili ya mahudhurio ya miadi zilikuwa na manufaa ya wastani ya muda mfupi kwenye ufubazaji wa virusi na hazikuathiri kubakia kwenye huduma au ufubazaji wa virusi baada ya kusitishwa. Matokeo haya yanaonesha haja ya kuelewa makundi madogo ambayo yatanufaika zaidi na motisha za kusaidia huduma za VVU.

Ufadhili: National Institute of Mental Health
